# Alfalfa modified the effects of degraded black soil cultivated land on the soil microbial community

**DOI:** 10.3389/fpls.2022.938187

**Published:** 2022-08-19

**Authors:** Linlin Mei, Na Zhang, Qianhao Wei, Yuqi Cao, Dandan Li, Guowen Cui

**Affiliations:** College of Animal Science and Technology, Northeast Agricultural University, Harbin, Heilongjiang, China

**Keywords:** soil degradation, phytoremediation, alfalfa, crop rotation, microbial community

## Abstract

Legume alfalfa (*Medicago sativa* L.) is extensively planted to reduce chemical fertilizer input to the soil and remedy damaged fields. The soil mechanism of these effects is potentially related to the variations in alfalfa-mediated interactions of the soil microbial community. To understand the impact of planting alfalfa on the soil microbial community in degraded black soil cultivated land, a 4-year experiment was conducted in degraded black soil cultivated land. We assessed soil parameters and characterized the functional and compositional diversity of the microbial community by amplicon sequencing that targeted the 16S rDNA gene of bacteria and ITS of fungi in four systems under corn cultivation at the Harbin corn demonstration base (Heilongjiang, China): multiyear corn planting (more than 30 years, MC1); 2 years of alfalfa-corn rotation (OC); 3 years of alfalfa planting (TA); and 4 years of alfalfa planting (FA). It was found out that alfalfa led to changes in the alpha diversity of soil bacteria rather than in fungi in the degraded arable land. The abundance of the bacterial groups Gemmatimonadetes, Actinobacteria, Planctomycetes, and Chloroflexi was increased in OC, while Proteobacteria and Acidobacteria and the fungal group Glomeromycota were increased in TA and FA. OC, TA, and FA significantly increased the pH level but reduced soil electrical conductivity, but they had no impact on soil available nitrogen and soil available potassium at the 0–15 cm soil depth. However, with the years of alfalfa planting, soil available nitrogen and soil available potassium were reduced at the 15–30 cm soil depth. OC, TA, and FA significantly reduced the soil available phosphorus and soil total phosphorus at the 15–30 cm soil depth. There was no significant impact made on soil total nitrogen. FA significantly reduced the soil organic matter at the 15–30 cm soil depth. Planting alfalfa in degraded black soil cultivated land can reduce the salt content of the soil, and the nutrient content of soil planted with alfalfa without fertilization was equivalent to that of degraded corn cultivated land with annual fertilization. Besides, alfalfa recruited and increased contained taxa with the capacity to improve soil nutrient utilization and inhibit the harmful influences of pathogens for subsequent crops. Meanwhile, the planting of alfalfa can modify soil conditions by promoting the proliferation of specific beneficial microbiota groups.

## Introduction

Black soil generally refers to Mollisols, which are the fertile soils distributed in four major regions around the world: North America, southeastern Europe, Central Asia, including northeast China, and the Pampas of South America (Durán et al., 2011; Liu et al., 2018a). However, in recent years, the black soil resources have been seriously degraded, the content of soil organic matter has decreased, the soil has hardened, and the degree of salinization has increased (Du et al., 2021; Li et al., 2021). In order to maintain a high grain yield, it is necessary to use a large amount of chemical fertilizers, which results in a large number of residual inorganic salt ions, thus exacerbating the degradation of the soil. The rotation of Gramineae and Leguminosae is a scientific and reasonable combination model for the restoration of degraded black soil.

Previous studies have shown that planting high-quality forage is an important measure to improve the quality and fertility of degraded arable soils (Yu et al., 2021). Meanwhile, a previous study observed improvements in field, pest management, and soil fertility with crop rotations. These improvements are attributed mainly to the soil microbial community (Samaddar et al., 2021).

Alfalfa has long been the preferred forage grass for rotation due to its high perennial persistence, protein content, and biomass production. Alfalfa is a potential “microbial hotspot crop” (Samaddar et al., 2021), as studies have revealed that planting alfalfa increases soil microbial activity (Agnello et al., 2016; Luo et al., 2018), and a large amount of root biomass and lipid root exudates closely related to plant growth and development can be produced (Wang et al., 2017; Bertrand et al., 2020; Zhao et al., 2020). In addition, the soil bacterial population can be introduced into the rhizosphere; for example, the addition of alfalfa in the rotation would increase the abundance of such bacterial groups as Acidobacteria, Actinobacteria, Chloroflexi, Gemmatimonadetes, and the fungal group Glomeromycota that include arbuscular mycorrhizal fungi (Wang et al., 2017; Samaddar et al., 2021). Planting alfalfa significantly improved the soil bacterial population and fungal/bacterial ratio after continuous potato cropping (Qin et al., 2017). A scientific and reasonable rotation mode can be effective in alleviating the deterioration of the soil environment caused by continuous cropping obstacles, enhancing the activity and diversity of microorganisms in soil, and creating a healthy and stable soil ecological environment for subsequent crop growth (Jarecki et al., 2018). Therefore, an improved understanding as to the mechanism of interaction between microorganisms and plants could help improve the remediation of degraded black soil.

Legume alfalfa also has the ability to improve the content of soil organic matter and nutrients (Beimforde et al., 2014; Dong et al., 2016; Luo et al., 2018). However, research suggested a decreasing trend in total nitrogen, total carbon, nitrate nitrogen, and available potassium in alfalfa for up to 10 years after continuous cropping, while the opposite was true for more than 10 years of continuous cropping (Yao et al., 2019). Meanwhile, the changes in the organic matter and nutrient content of the soil after alfalfa cultivation can affect the structure of soil fungal community and the relative abundance of specific fungi (Shen et al., 2013; Bastida et al., 2017; Yao et al., 2019). Research has demonstrated that alfalfa continuous cropping will accelerate the depletion of soil water and phosphorus, thus reducing bacterial diversity (Beauregard et al., 2010; Wang et al., 2021). However, there remain many uncertainties with regard to the impact of alfalfa planting on the soil microbial community and the restoration of degraded black soil.

Herein, our aim is to investigate the impact of planting alfalfa on the soil microbial community in degraded black soil cultivated land. Thus, we drew comparison between multiyear corn planting (more than 30 years), 2 years of alfalfa-corn rotation, 3 years of alfalfa planting, and 4 years of alfalfa planting in all parts of cropping system experiment. It was predicted that (i) alfalfa recruits and increases the microbial community, including taxa with the capacity to improve soil nutrient utilization and promote luxuriant growth for subsequent crops, and (ii) long-term alfalfa planting can modify soil conditions by promoting the proliferation of specific beneficial microbiota groups.

## Materials and methods

### Experimental design

Conducted at the Harbin corn demonstration base, the research is a long-term agricultural experiment carried out at Wujia Town (126°23′E, 45°31′N) in Heilongjiang Province. The annual average temperature and precipitation are 3.5°C–4.5°C and 400–600 mm, respectively. The soil type of the experimental site is black soil. The cultivars of corn and alfalfa used were “Tiannong 9” and “Dongnong 1,” respectively. A potassium sulfate compound fertilizer substrate of 600 kg·hm^−2^ (N 72 kg·hm^−2^, P 108 kg·hm^−2^, K 90 kg·hm^−2^) was applied annually to the corn field. The alfalfa was sown in May 2016 and 2017, respectively. The alfalfa fields were fertilized in the year of sowing with 54 kg·hm^−2^ N and 138 kg·hm^−2^ P. Only a foliar spray was applied 10 days before each crop was mown during the growth of alfalfa.

The four treatments included: multiyear corn planting (more than 30 years, MC1); 2 years of alfalfa corn rotation (OC), where corn was cultivated following two-year alfalfa in 2019; 3 years of alfalfa planting (TA); and 4 years of alfalfa planting (FA). Each treatment was replicated 4 times. The size of each plot was 20 m × 20 m, with 5 m between plots. The topography of the plots was flat, and the soil quality was uniform.

### Sampling and laboratory analyses

Soil samples were collected in mid-September 2019 (after crop harvesting). Within each plot, 10 sample points were randomly selected with a 2.5 cm diameter soil auger; then, the soils were mixed into one sample. The root system of alfalfa was a typical taproot system purposed to study the effect of alfalfa on different soil layers for each treatment, with samples collected from two soil depths: 0–15 cm and 15–30 cm. Within each plot, 10 sample points were randomly selected with a 2.5 cm diameter soil auger. Then, the soils were mixed into one sample. Then, the samples were sieved and divided into two parts. One part was air-dried for soil parameters determination, while the other was reserved at −80°C for microbial analysis.

After the soil surface litter was removed, the soil samples were air-dried at room temperature and then passed through a 2 mm soil sieve for the determination of soil parameters. The soil bulk density (BD) was measured by using the ring knife method. The soil moisture content (MC) was tested by the weight loss of the samples after drying at 105°C. Soil pH and electrical conductivity (EC) were measured using a pH meter and a conductivity meter, respectively (water-soil ratio was 2.5:1; Han et al., 2018). Soil total nitrogen was tested by using the Kjeldahl method. After NaOH melting, soil total nitrogen (TN), soil total phosphorus (TP), and soil total potassium (TK) were tested by means of Kjeldahl nitrogen determination, molybdenum antimony colorimetry, and flame photometry, respectively. Soil available nitrogen (AN) was tested by using the alkaline diffusion method. Soil available phosphorus (AP) was tested by using the NaHCO_3_ extraction-molybdenum antimony anti-colorimetric method. Soil available potassium (AK) was extracted with neutral ammonium acetate and tested with a flame photometer. Soil organic matter (SOM) was tested by means of oxidation with potassium dichromate-sulfuric acid solution (Sun, 2019).

### Amplicon sequencing

The diversity and composition of the soil bacterial and fungal communities were determined by Dene Denovo from Guangzhou. We amplified the V3–V4 region of bacterial 16S rDNA and the ITS2 region of fungal ITS rDNA by using specific primer pairs with barcodes 341F: 5′CCTACGGGNGGCWGCAG3′/806R: 5′ GGACTACHVGGGTATCTAAT3′ and ITS3_KYO2: 5′ GATGAAGAACGYAGYRAA3′/ITS4: 5′ TCCTCCGCTTATTGATATGC3′. Each sample was subjected to PCR in a 50 μl mixture. The mixture included 10× Buffer KOD (5 μl), 2 mM dNTPs (5 μl), 25 mM MgSO_4_ (3 μl), KOD Polymerase (1 μl), 10 μΜ Primer F (1.5 μl), 10 μM Index Primer (1 μl), 10 μM Primer R (1.5 μl), or 10 μM Universal PCR Primer (1 μl). The reaction conditions for the PCR are as follows: 94°C (2 min), 98°C (10 s), 65°C (30 s), and a final extension at 68°C (10 min). Then, all raw tags were filtered into sequences to obtain high-quality tags. In order to study the compositional diversity information on the species of the samples, all valid tags of all samples were clustered using UPARSE software. Then, the sequences were clustered into OTUs (Operational Taxonomic Units) with 97% agreement by default. The results were calculated and the absolute abundance and relative information of tags in each sample were calculated for each OTU. UPARSE selects representative sequences (the tag sequence with the highest abundance in OTUs) in the process of constructing OTUs. The Naive Bayesian assignment algorithm of RDP Classifier was applied to annotate these representative sequences with the Greengenes database. These representative sequences were then annotated with the Greengenes database using the Naive Bayesian assignment algorithm of RDP Classifier (with a confidence threshold of 0.8~1), so as to obtain the species annotation information of each OTU.

### Statistical analysis

Soil parameters, Chao1, Shannon, and Sob were determined by means of one-way ANOVA, PCA, and PERMANOVA tests. The community composition at the OTU level of soil microorganisms was analyzed through PCoA (using Bray–Curtis distances) and PERMANOVA tests. Pearson’s correlation analysis was conducted to identify soil physicochemical factors and determine the relative abundance of bacterial and fungal phyla. All data analyses and graphing were performed with the assistance of SPSS 26 and Origin 2019b, respectively. The comparisons between the means were performed through Tukey’s test (*p* < 0.05).

## Results

### Effects on the composition of soil bacterial and fungal communities

**Figure 1:**
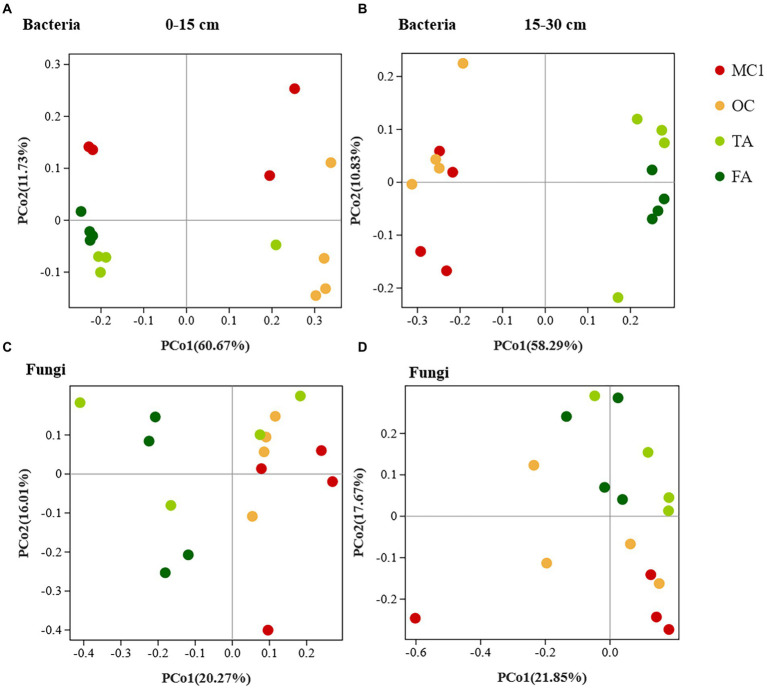
The PCoA of soil bacteria and fungals at different soil depths. The PCoA of soil bacteria at the 0-15 cm soil depth **(A)** and the 15-30 cm soil depth **(B)**. PCoA of soil fungi at the 0-15 cm soil depth **(C)** and the 15-30 cm soil depth **(D)**.

The changes in planting practices affected the bacterial and fungal communities. At the 0–15 cm soil depth, the bacterial and fungal communities were generally clustered by sample point (Figures 1A,B). Besides, PERMANOVA showed that the soil bacterial and fungal communities of TA, FA, and OC clearly differed from those of MC1 (*p* = 0.004, *p* = 0.001). At the 15–30 cm soil depth, soil bacterial communities differed significantly between corn and alfalfa plantings, while the closer distance between OC and MC1 along axis 1 indicated the slight differences (Figure 1C). Also, PERMANOVA indicated that they were insignificant (*p* = 0.181). The distance between TA and FA was small, while the two bacterial communities were similar and insignificantly different (*p* = 0.543). The fungal communities were clustered at different sites. The differences in fungal communities between maize and alfalfa plots were observed along axis 1 for years of cultivation, with closer distances between samples from three-year alfalfa and four-year alfalfa plots, indicating that short-term alfalfa cultivation exerted no significant effect on fungal community composition (Figure 1D). PerMANOVA showed that the soil fungal communities differed significantly (*p* = 0.005) between the plantings.

### Alpha diversity of soil bacterial and fungal communities

We detected a total of 3,265,694 (94.87% of the total community) effective tags of bacteria from 32 soil samples, with each ranging from 302 to 478 in length. A total of 80,163 OTUs were detected through clustering. Moreover, we detected 3,310,770 (95.95% of the total community) fungal active labels from 32 soil samples, with each ranging from 206 to 478 in length. These tags were then clustered into 24,894 OTUs.

**Figure 2:**
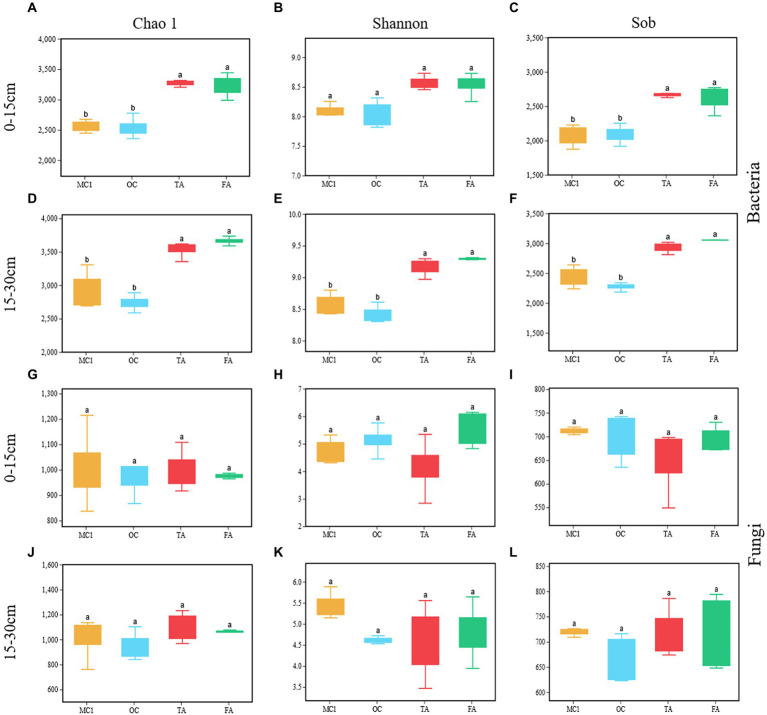
Box plots representing soil bacterial **(A-F)** and soil fungal **(G-L)** alpha diversity at the soil depths of 0-15 cm and 15-30 cm for different treatments, including Chao1, Shannon and Sob. MC1= multiyear corn planting. OC = two years of alfalfa-corn rotation, TA = three years of alfalfa planting, and FA= four years of alfalfa planting. Different lowercase letters indicate a significant difference between treatments (*p* < 0.05).

OC has no significant impact on Chao 1, Shannon, and sobs (Figures 2A–L). TA and FA significantly increased the Chao 1 and Sobs indices of the soil bacterial community at the soil depths of 0–15 cm and 15–30 cm (*p* < 0.05; Figures 2A,C,D,F). FA and TA significantly increased the Shannon index at the 15–30 cm soil depth (*p* < 0.05; Figure 2E). There were no significant impacts made on the Chao 1, Sobs, and Shannon indices of the soil fungal community (Figures 2G–L).

### Overall microbial composition patterns

**Figure 3:**
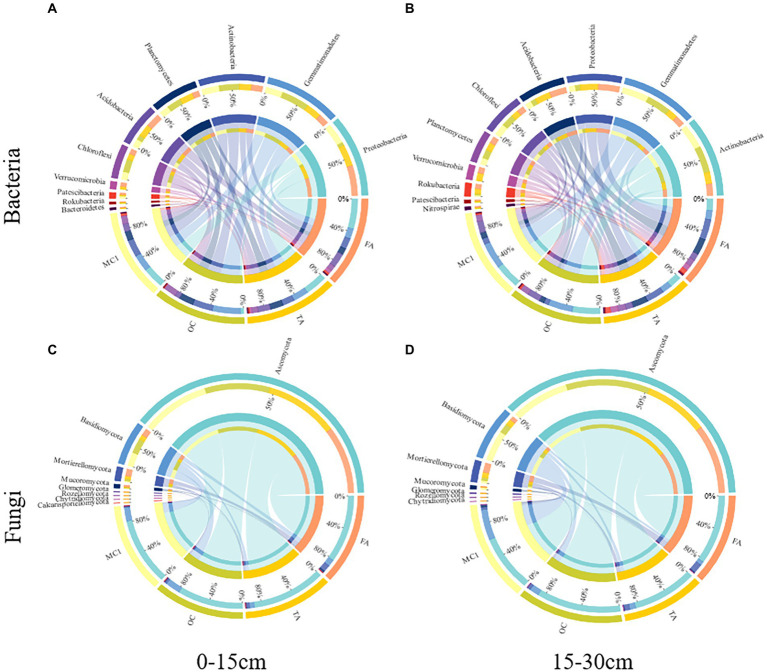
Circos plots represent the relative abundance of the top 10 bacterial phyla and the top 8 fungal phyla at different soil depths. The relative abundance of bacteria at the 0-15 cm soil depth **(A)** and the 15-30 cm soil depth **(B)** and the relative abundance of fungi at the 0-15 cm soil depth **(C)** and the 15-30 cm soil depth **(D)** under different treatments.

A total of 31 bacterial phyla and 10 fungal phyla were detected (Figure 3). At the 0–15 cm soil depth, OC increased the abundance of Gemmatimonadetes, Actinobacteria, Planctomycetes and Chloroflexi by 62.91%, 16.11%, 52.93%, and 62.03% (*p* < 0.05; Figure 3A), respectively. FA increased the abundance of Proteobacteria by 44.52% (*p* < 0.05). TA and FA increased the abundance of Acidobacteria by 56.83% and 81.06% (*p* < 0.05), respectively, with the abundance of Verrucomicrobia improved by 204.45% and 416.06% (*p* < 0.05), respectively; however, they reduced the abundance of Gemmatimonadetes by 10.17% and 38.70% (*p* < 0.05), respectively, with the abundance of Actinobacteria increased by 31.93% and 35.69% (*p* < 0.05), respectively, with the abundance of Chloroflexi improved by 24.11% and 36.31% at the 0–15 cm soil depth (*p* < 0.05; Figure 3A), respectively. TA and FA increased the abundance of Proteobacteria by 191.38% and 241.87% (*p* < 0.05), respectively, with the abundance of Acidobacteria improved by 149.02% and 168.44% (*p* < 0.05), respectively, with the abundance of Planctomycetes increased by 20.12% and 2.51% (*p* > 0.05), respectively; by contrast, the abundance of Actinobacteria was reduced by 52.17% and 52.45% (*p* < 0.05), respectively, with the abundance of Gemmatimonadetes improved by 63.92% and 64.73% (*p* < 0.05), respectively, with the abundance of Chloroflexi reduced by 40.47% and 42.93% at the 15–30 cm soil depth (*p* < 0.05; Figure 3B), respectively.

At the 0–15 cm soil depth, OC, TA, and FA increased the abundance of Mortierellomycota by 126.80%, 216.84%, and 645.29% (*p* < 0.05), respectively, with the abundance of Mucoromycota increased by 130.03%, 107.81%, and 414.69% (*p* < 0.05; Figure 3C). TA and FA increased the abundance of Glomeromycota by 35.78% and 372.35% (*p* < 0.05; Figure 3C), respectively. At the 15–30 cm soil depth, OC, TA, and FA increased the abundance of Ascomycota by 43.75%, 10.22%, and 22.86% (*p* < 0.05; Figure 3D), respectively.

**Figure 4:**
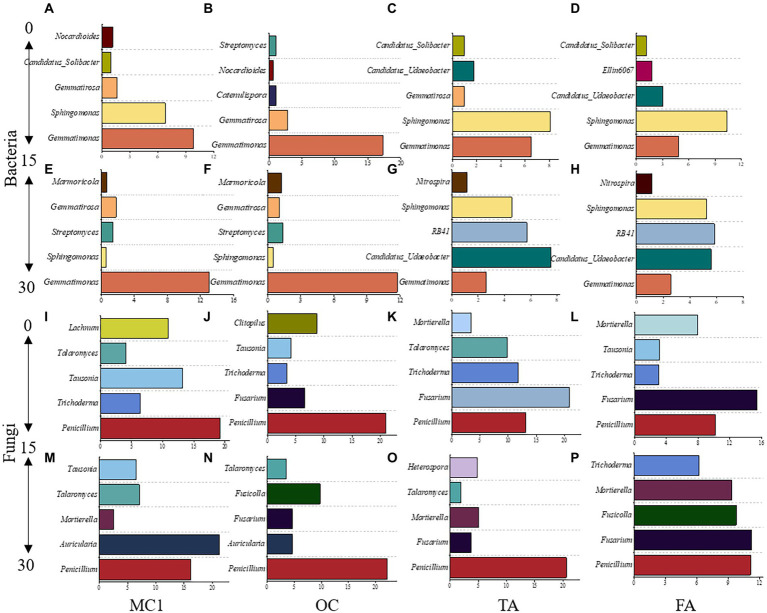
The top five dominant genera at the soil depths of 0-15 cm and 15-30 cm under different treatments. The dominant bacterial genera at the 0-15 cm soil depth **(A-D)** and 15-30 cm soil depth **(E-H)**. The dominant fungal genera at the 0-15 cm soil depth **(I-L)** and the 15-30 cm soil depth **(M-P)**.

To gain insight into the community differentiation of soil microbial taxa after alfalfa planting, the five most abundant genera were observed at different soil depths. For bacteria, *Gemmatimonas* was dominant at both soil depths. The next most abundant genera were *Sphingomonas* in the 0–15 cm soil depth and *Candidatus_Udaeobacter* and *RB41* in the 15–30 cm soil depth (Figures 4A–H). *Nitrospira* was observed in TA and FA at the 15–30 cm soil depth (Figures 4E,F).

*Gemmatimonas* was the highest at 0–15 cm and 15–30 cm soil depth in MC1 and OC (Figures 4A,B,E,F). *Sphingomonas* was the highest at 0–15 cm in TA and FA (Figures 4C,D). For fungi, *Penicillium* was the highest at both soil depths under the different treatments. *Fusarium* and *Mortierella* were the dominant species at both soil depths in TA and FA (Figures 4I–P).

### Soil parameters

The physicochemical properties of the soil varied considerably when different planting methods were adopted. TA and FA significantly reduced the soil moisture content but increased the soil bulk density at the 0–15 cm soil depth. OC, TA, and FA significantly increased the pH value but reduced soil electrical conductivity at the 0–15 cm soil depth. OC, TA, and FA had no impact on soil available nitrogen and soil available potassium at the 0–15 cm soil depth. However, with the extension of alfalfa planting, soil available nitrogen and soil available potassium were reduced at the 15–30 cm soil depth. OC, TA, and FA significantly reduced the soil available phosphorus and soil total phosphorus at the 15–30 cm soil depth. There was no significant impact on soil total nitrogen. FA significantly reduced the soil organic matter at the 15–30 cm soil depth (Table 1).

**Table 1:** Effect of planting practices on soil physicochemical properties.

Soil depth cm	Sample	MC%	BD g cm^−3^	pH	EC μs cm^−1^	AN mg kg^−1^	AP mg kg^−1^	AK mg kg^−1^	TN g kg^−1^	TP g kg^−1^	TK g kg^−1^	SOM g kg^−1^
0–15	MCI	26.74 ± 0.40a	1.06 ± 0.03b	5.92 ± 0.06b	81.70 ± 15.52a	111.42 ± 7.88a	89.36 ± 5.30a	103.75 ± 5.79ab	1.35 ± 0.03a	0.66 ± 0.02a	20.48 ± 0.69ab	28.79 ± 2.92a
	OC	26.32 ± 0.40a	1.12 ± 0.06b	6.33 ± 0.06a	45.58±6.98b	120.75 ± 6.64a	34.98 ± 4.70b	100.83 ± 4.90b	1.29 ± 0.04a	0.47 ± 0.05b	24.33 ± 1.46a	27.83 ± 2.48a
	TA	24.84± 0.65bc	1.35 ± 0.05a	6.31 ± 0.01a	34.98 ± 1.65a	102.20 ± 9.611a	34.93 ± 3.92b	127.33 ± 6.31a	1.38 ± 003a	0.39 ± 0.05b	18.26 ± 0.87b	28.32 ± 1.00a
	FA	24.44 ± 0.22b	1.40 ± 0.02a	6.28 ± 0.01a	32.13 ± 0.65b	88.67 ± 7.57a	30.35 ± 1.93b	116.17 ± 6.63ab	1.28 ± 0.02a	0.38 ± 0.05b	19.47 ± 0.32b	27.02 ± 1.31a
15–30	MCI	26.07 ± 0.29a	1.20 ± 0.02b	6.63 ± 0.13a	48.45 ± 7.69a	111.07 ± 6.15ab	30.06 ± 5.27a	133.50 ± 1.79a	1.18 ± 0.02ab	0.44 ± 0.03a	19.78 ± 0.75b	27.46 ± 0.79a
	OC	26.00 ± 0.77a	1.04 ± 0.04c	6.88 ± 0.05a	48.75 ± 5.97a	120.05 ± 3.53a	28.65 ± 6.15a	118.42 ± 1.97b	1.28 ± 0.05a	0.42 ± 0.05a	24.18 ± 1.29a	28.47 ± 1.25a
	TA	25.35 ± 0.54a	1.31 ± 0.02ab	6.76 ± 0.02a	35.23 ± 1.40a	97.71 ± 2.85b	17.87 ± 6.23a	92.58 ± 2.69c	1.23 ± 0.03ab	0.37 ± 0.02a	18.02 ± 1.05b	24.30 ± 1.16ab
	FA	24.72 ± 0.13a	1.39 ± 0.05a	7.00 ± 0.15a	47.43 ± 11.87a	76.42 ± 3.57a	16.57 ± 2.83a	88.17 ± 3.22c	1.10 ± 0.05b	0.31 ± 0.01a	19.34 ± 0.12b	22.22 ± 1.39b

Mean ± standard errors are shown in the table. Different letters indicate significant differences (Tukey’s test, *P* < 0.05).

**Figure 5:**
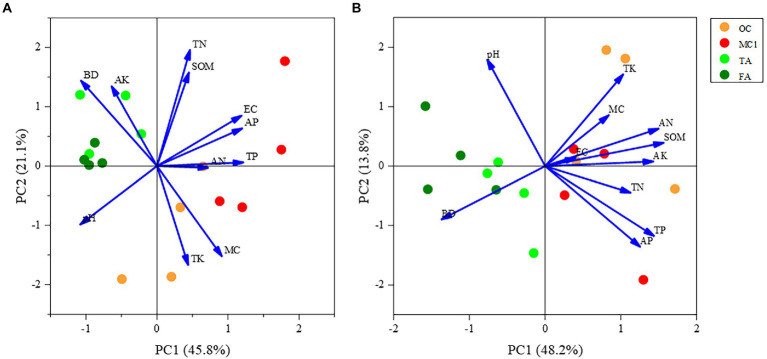
PCA of soil physicochemical properties. The PCA of soil physicochemical properties at the 0-15 cm soil depth **(A)** and soil physicochemical properties at the 15-30 cm soil depth **(B)**.

At the 0–15 cm soil depth, PC1 explained 45.8% of the variation, and 66.9% of the variation in total with PC2. As shown in the figure, there was a more pronounced separation between the corn and alfalfa plots along the first sorting axis, indicating that the physicochemical properties differed substantially between the OC and TA. Soil pH and soil bulk density were negatively correlated with planting method along the first ranking axis, but positively correlated with soil total phosphorus, soil available phosphorus, and soil electrical conductivity. Soil available potassium, soil organic matter, and soil available nitrogen show shorter arrows, indicating a less significant impact on the sorting plane. The distance between MC1 and OC sample sites was increased, indicating that the change in cropping method (2 years of alfalfa planting followed by maize planting in a multi-year maize field) caused a significant difference in soil physicochemical properties between them. Also, the difference between TA and FA is insignificant (Figure 5A).

At the 15–30 cm soil depth, PC1 explained 48.2% of the variation, and PC2 explained 13.8% of the variation, according to the principal component analysis. A total of 62% of the variation was explained by the principal component analysis. As can be seen from the figure, the physicochemical properties of the corn field along the first sorting axis were significantly different from those of the alfalfa field. Additionally, the planting method was positively correlated with soil available potassium and soil organic matter. There was a significant separation between MC1 and OC along the second ordination axis, indicating that the soil physicochemical properties differed significantly due to the change in cropping practice. However, the difference between the physicochemical properties of TA and FA was less significant (Figure 5B).

### Relationships between soil properties and microbial taxa

**Figure 6:**
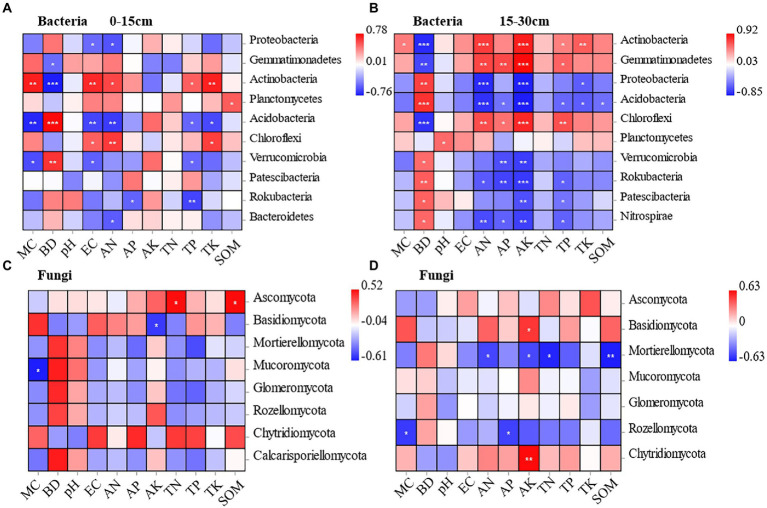
Pearson correlation between soil bacteria and soil physicochemical properties at the depths of 0-15 cm and 15-30 cm under different treatments **(A, B)**. Pearson correlation between soil fungal and soil physicochemical properties at the depths of 0-15 cm and 15-30 cm under different treatments **(C, D)**.

At the 0–15 cm soil depth, Proteobacteria and Acidobacteria were negatively correlated with soil electrical conductivity and soil available nitrogen to a significant extent under different planting methods. Actinobacteria and Chloroflexi were positively correlated with soil electrical conductivity and soil available nitrogen to a significant extent. In addition, Acidobacteria and Rokubacteria showed a significant negative correlation with soil total phosphorus. Meanwhile, Acidobacteria (r = −0.735) and Verrucaria (r = −0.6) were negatively correlated with soil moisture content to a significant extent, and Actinobacteria (r = 0.716) showed a significant positive correlation with soil moisture content. Gemmatimonadetes (r = −0.50) and Actinobacteria (r = −0.765) were negatively correlated with soil bulk density to a significant extent, while Acidobacteria (r = 0.779) and Verrucomicrobia (r = 0.65) were positively correlated with soil bulk density to a significant extent. Rokubacteria was negatively correlated with soil total phosphorus and soil available phosphorus to a significant extent. The Planctomycetes was positively correlated only with soil organic matter to a significant extent (Figure 6A).

At the 15–30 cm soil depth, Gemmatimonadetes (r = 0.713, r = 0.923), Actinobacteria (r = 0.747, r = 0.849), and Chloroflexi (r = 0.681, r = 0.818) were positively correlated with soil available nitrogen and soil available potassium to a significant extent. While, Proteobacteria (r = −0.799, r = −0.839), Acidobacteria (r = −0.778, r = −0.854), and *Nitrospira* (r = −0.678, r = −0.721) were negatively correlated with soil available nitrogen and soil available potassium to a significant extent. Gemmatimonadetes (r = −0.694), Actinobacteria (r = −0.802), and Chloroflexi (r = −0.77) were negatively correlated with soil bulk density to a significant extent. Proteobacteria (r = 0.726) and Acidobacteria (r = 0.779) were positively correlated with soil bulk density to a significant extent. Unlike the 0–15 cm soil depth, soil available phosphorus was associated with more bacterial communities. Gemmatimonadetes (r = 0.697) was positively correlated with soil available phosphorus to a significant extent, while Verrucomicrobia (r = −0.660), and Rokubacteria (r = −0.705) were negatively correlated with soil available phosphorus to a significant extent (Figure 6B).

The correlations between fungal community composition and soil factors under different cultivation methods were examined by conducting Pearson’s correlation analysis. According to the results, soil physicochemical properties had significant effects on the relative abundance of fungal phylum, and the effects varied by soil depth. At the 0–15 cm soil depth, Mucoromycota was negatively correlated with soil moisture content to a significant extent (r = −0.610). Basidiomycota was negatively correlated with soil available potassium to a significant extent (r = −0.536). Ascomycota was positively correlated with soil total nitrogen (r = 0.513) and soil organic matter (r = 0.521) to a significant extent (Figure 6C). It indicates that the dominant soil fungal taxa Ascomycota and Basidiomycota are significantly influenced by environmental factors. At the 15–30 cm soil depth, Basidiomycota and Chytridiomycota were positively correlated with soil available potassium to a significant extent (r = 0.515, r = 0.628). Mortierellomycota was negatively correlated with soil available nitrogen (r = −0.515), soil available potassium (r = −0.509), soil total nitrogen (r = −0.606), and soil organic matter (r = −0.626), showing a significant negative correlation. Rozellomycota was negatively correlated with soil moisture content (r = −0.547) and soil available phosphorus (r = −0.529) to a significant extent (Figure 6D).

## Discussion

Alfalfa led to changes in the alpha diversity of soil bacteria rather than in fungi in the degraded arable land. Adding alfalfa to the rotation recruited and increased the microbial community, including taxa with the capacity to improve soil nutrient utilization and promote luxuriant growth for subsequent crops, such as Gemmatimonadetes, Actinobacteria, Planctomycetes, and Chloroflexi. Long-term alfalfa planting can modify soil conditions by promoting the proliferation of specific beneficial microbiota groups like Proteobacteria and Acidobacteria. Planting alfalfa in degraded black soil cultivated land can help reduce the salt content of the soil, and the nutrient content of soil planted with alfalfa without fertilization was equivalent to that of degraded corn cultivated land with annual fertilization.

Corn and alfalfa rotation makes no significant impact on the alpha diversity of soil bacteria and fungi. However, planting alfalfa for 3 or 4 years led to variations in the alpha diversity of soil bacteria rather than in fungi in the degraded arable land, which is consistent with the outcomes of a previous research (Szoboszlay et al., 2017; Luo et al., 2018). Corn is fertilized twice a year, and the long-term application of chemical fertilizers can cause soil organic matter to accumulate (Tejada and Gonzalez, 2008). The roots of corn stubble remain in the soil after corn harvest, while corn residues and secretions can maintain the organic carbon balance (Bajgai et al., 2014). However, due to the destruction of soil aggregates by farming, the microhabitat is lost, which rearranges the spatial environment of the microbial community, promotes soil homogenization, and reduces microbial groups (Lupwayi et al., 2001; Acosta-Martínez et al., 2010). Meanwhile, alfalfa has developed roots, which can go deep into the lower layer of the soil and exert a strong fixation force on the soil, which results in a large rhizosphere area, thus providing nutrients for soil bacteria and increasing the density of bacteria (Solly et al., 2014).

In the present study, the alpha diversity indices of soil fungi remained consistent, and there was no significant difference observed among corn fields, corn alfalfa rotation, and alfalfa planting. In spite of this, at the 15–30 cm soil depth, all diversity indices of the TA and FA fungal communities were found slightly higher than those of MC1. The results demonstrate that planting alfalfa is conducive to the proliferation of fungi under certain conditions, but this process may be a lengthy one. In a study, it has been reported that the long-term continuous cropping of alfalfa (more than 6 years) increased fungal diversity (Yao et al., 2019), which is consistent with our results. In addition, fertilization affects the structure of the soil microbial community and may lead to soil mycosis, such as *Fusarium* (Beauregard et al., 2010). Herein, it was found out that although the alfalfa field is no longer fertilized after planting, its roots can still increase organic matter and nodule nitrogen fixation for the soil, maintain the number of soil fungi and slightly improve the diversity, which is conducive to the survival of fungi. Moreover, corn and alfalfa rotation and planting of alfalfa for 3 and 4 years led to the appearance of plant growth-promoting fungi *Trichoderma* at 0–15 cm soil depth.

In the natural soil, the fungal community in the rhizosphere of maize is similar to that in the original soil, with most of the fungi in the rhizosphere stemming from the soil. There were similar major bacterial phyla after adding alfalfa to the rotation, but the relative abundance of dominant taxa changed significantly. OC increased the abundance of Gemmatimonadetes, Actinobacteria, Planctomycetes, and Chloroflexi, which are contained in nutrient cycling, organic matter turnover, and plant growth-promoting activities, at the 0–15 cm soil depth. Plant microbial interactions and specific groups outside of larger soil communities are recruited by plants (Berendsen et al., 2012). The microorganisms colonizing the alfalfa rhizosphere would be carried over during alfalfa and corn rotation (Samaddar et al., 2021). Adding legume to crop rotation can enrich the genes related to nitrogen cycle in the rhizosphere of subsequent crops, with legume rhizosphere microorganisms left behind.

Meanwhile, the abundance of Proteobacteria and Acidobacteria was increased by TA and FA at the soil depths of 0–15 cm and 15–30 cm. Proteobacteria prefer environments with high effective carbon contents (Ren et al., 2018) and play an important role in saline-alkali soil remediation, pollutant degradation, and plant pest control (Broszat et al., 2014). Alfalfa is a legume forage. The nodule structure can significantly improve biomass accumulation. After the planting of alfalfa, the growth and physiological activity of the root system increase. Root exudates are conducive to promoting the reproduction of soil microorganisms and making Proteobacteria directly related to the rhizosphere effect. Acidobacteria are often more abundant in soils with low resource availability (Liu et al., 2014; Kielak et al., 2016). In the present study, with the extension of alfalfa planting, soil moisture content decreased, which is consistent with the survival strategy of Acidobacteria. Meanwhile, due to the lack of fertilization in alfalfa fields and the reduction in soil nutrients, it provides suitable conditions for its survival series. Therefore, the relative abundance increases. However, the reduced abundance of Gemmatimonadetes, Actinobacteria, and Chloroflexi in the following alfalfa is likely to result from the difference in tillage regimes. In addition, the dominant taxa of soil bacteria were more significantly affected by soil parameters, with Actinobacteria and Chloroflexi being more sensitive to the variations in environmental factors than other dominant taxa.

The relative abundance of Ascomycota also showed no significance but a slightly elevated difference. Fungi are not sensitive to aboveground vegetation changes and farming patterns, and fewer soil physicochemical factors are significantly associated with the relative abundance of the fungal phylum, which is consistent with previous research results (Hibbett et al., 2007). The abundance of Glomeromycota was significantly increased by TA and FA. Glomeromycota has arbuscular mycorrhizal (AM) fungi, which can form a reciprocal symbiosis with plants (Mei et al., 2019). With the extension of alfalfa planting, AM fungi abundance increased, which may be caused by reduced soil disturbance. These results suggest that the long-term planting of alfalfa will improve the colonization and activity of AM fungi in the soil system (Samaddar et al., 2021).

One of the functions performed by planting alfalfa is to accumulate nitrogen reserves for soil, increase the mineralization rate, and improve nitrogen availability under soil microbial activity. In this study, *Nitrospira* was increased, which is an aerobic chemolithoautotrophic nitrite-oxidizing bacteria (Stein, 2019) in the TA and FA. Meanwhile, a greater abundance of S*phingomonas* was observed in TA and FA. *Sphingomonas* has a wide range of metabolic capacities, some strains of which can synthesize valuable extracellular biopolymers and degrade harmful substances in soil. It is thus applicable as a bacterial antagonist of plant pathogenic fungi (Feng et al., 2021). Moreover, the main flora of fungi also changed when different planting methods were applied. As a microbial fertilizer, *Trichoderma* can inhibit most soil-borne pathogens (Bidellaoui et al., 2019) and appear in corn and alfalfa rotation in TA and FA. Plant growth-promoting rhizobacteria *Mortierella* appeared in TA and FA. Our results demonstrate that alfalfa recruits and increases specific symbiotic microbes to adapt and modify degraded black soil cultivated land with the long-term application of chemical fertilizer for a better chance of survival. However, we also detected the pathogenic fungal genus *Fusarium*, which causes plant diseases (Samaddar et al., 2021), but disease incidence was not observed in corn and alfalfa. At present, the reason for alfalfa selective aggregation *Fusarium* remains unclear, which requires verification in the future.

Soil parameters affect plant growth and soil microorganisms. In the present study, TA and FA reduced soil moisture content, soil electrical conductivity, soil available phosphorus, and soil total phosphorus at the 15–30 cm soil depth (Huang et al., 2018; Cao et al., 2021), which is consistent with our finding that TA and FA reduced soil moisture content. Meanwhile, alfalfa increased pH value and reduced soil electrical conductivity. These results show that alfalfa can not only neutralize the pH level of acidic soil and reduce the salt content of soil (Zhao et al., 2016; Liu et al., 2018b), but also promote the development of the soil microbial community and improve soil quality.

Although alfalfa has high requirements for soil phosphorus, the phosphorus in the soil is consumed with the extension of planting, which reduces the contents of soil available phosphorus and soil total phosphorus in the soil after alfalfa planting.

Gramineae corn was a fibrous root system, the root of which is slender and can go deep into the soil layer to absorb nutrients, especially calcium and phosphorus. However, in this process, Gramineae plants lack nitrogen and need to be supplemented by legume plants with deep roots. In this study, corn and alfalfa rotation increased soil available nitrogen and soil total nitrogen. Alfalfa in rotations can improve nitrogen in the soil of subsequent crops. Moreover, planting alfalfa made no difference to soil available nitrogen, soil available potassium, soil total nitrogen, or soil organic matter. Alfalfa nitrogen is derived from the rhizobial nitrogen fixation (Xie et al., 2015) on the degraded black soil cultivated land where corn has been planted for a long time, and the nutrient content of soil (soil total nitrogen, soil total potassium, and soil organic matter) planted with alfalfa without fertilization is equivalent to that of degraded corn cultivated land with annual fertilization. A previous research demonstrated that the reclamation of native sandy grasslands into alfalfa fields significantly increased soil organic carbon, total nitrogen (Beimforde et al., 2014), and available phosphorus contents (Dong et al., 2016). Pot experiments have also shown that planting alfalfa significantly enhanced soil nutrition (Luo et al., 2018). Therefore, planting alfalfa can improve the degraded black soil cultivated land and reduce the use of chemical fertilizer. Compared with the planting of alfalfa for 3 years, soil nutrients decreased slightly after planting alfalfa for 4 years. Besides, with the years of alfalfa planting, soil available nitrogen and soil available potassium were reduced at the 15–30 cm soil depth. Therefore, planting alfalfa for 3 years is more effective in improving soil fertility.

## Conclusion

Our results demonstrated that the alfalfa in rotations recruits and increases the contained taxa with the capacity to improve soil nitrogen and inhibit the detrimental impacts of pathogens on subsequent crops. Alfalfa continuous cropping can modify soil conditions by promoting the proliferation of specific beneficial microbiota groups. The nutrient content of soil planted with alfalfa instead of fertilization is equivalent to that of degraded corn cultivated land with annual fertilization. Meanwhile, alfalfa increased the pH level of acid soil, suggesting that alfalfa may have the potential to improve the degraded black soil on cultivated land and reduce the use of chemical fertilizer. However, with the years of alfalfa planting, soil available nitrogen and soil available potassium were reduced at the 15–30 cm soil depth. Planting alfalfa for 3 years exerts a better effect on improving soil fertility. Our study revealed the effects of alfalfa (rotation and continuous cropping) on soil microbial community structure and soil nutrients of degraded black soil cultivated land, thus providing a basis for the in-depth understanding of soil microbial groups and contributing to the restoration of degraded black soil cultivated land. In the future, it is necessary to advance the potential of soil microorganisms and produce potential biological inoculants by conducting an additional research on microbial community functional character through meta-proteome analysis or meta-transcriptome.

## Data availability statement

The raw data supporting the conclusions of this article will be made available by the authors, without undue reservation.

## Author contributions

LM and NZ performed the data analyses and wrote the manuscript. QW and YC contributed significantly to the analysis and manuscript preparation. DL performed the experiment. GC contributed to the conception of the study. All authors contributed to the article and approved the submitted version.

## Funding

This work was financially supported by Forestry Science and Technology Extension Demonstration Project of central Finance (TG 02), the China Postdoctoral Science Foundation (2021M690575), and Young Talents Project of Northeast Agricultural University (20QC17).

## Conflict of interest

The authors declare that the research was conducted in the absence of any commercial or financial relationships that could be construed as a potential conflict of interest.

## Publisher’s note

All claims expressed in this article are solely those of the authors and do not necessarily represent those of their affiliated organizations, or those of the publisher, the editors and the reviewers. Any product that may be evaluated in this article, or claim that may be made by its manufacturer, is not guaranteed or endorsed by the publisher.
